# The anti-diabetic effects of NAG-1/GDF15 on HFD/STZ-induced mice

**DOI:** 10.1038/s41598-021-94581-y

**Published:** 2021-07-22

**Authors:** Pattawika Lertpatipanpong, Jaehak Lee, Ilju Kim, Thomas Eling, Seung Yeon Oh, Je Kyung Seong, Seung Joon Baek

**Affiliations:** 1 Laboratory of Signal Transduction, College of Veterinary Medicine and Research Institute for Veterinary Science, Seoul National University, 1 Gwanak-ro, Gwanak-gu, Seoul, 08826 South Korea; 2 National Institute of Environmental Health Science, 111 TW Alexander Dr. Research Triangle Park, NC 27709 USA; 3 Laboratory of Developmental Biology and Genomics, BK21 Plus Program for Advanced Veterinary Science, Research Institute for Veterinary Science, College of Veterinary Medicine, and Korea Mouse Phenotyping Center, Seoul National University, Seoul, 08826 South Korea; 4 Interdisciplinary Program for Bioinformatics, Seoul National University, Seoul, 08826 South Korea

**Keywords:** Metabolism, Molecular biology

## Abstract

Nonsteroidal anti-inflammatory drug-activated gene-1 (NAG-1) plays a role in various diseases. Here, the anti-diabetic effects of NAG-1 were evaluated using a high-fat diet/streptozotocin-induced diabetic mouse model. NAG-1-overexpressing transgenic (NAG-1 Tg) mice exhibited lower body weight, fasting blood glucose levels, and serum insulin levels than wild-type (WT) mice. The homeostatic model assessment of insulin resistance scores of NAG-1 Tg mice were lower than those of WT mice. Hematoxylin and eosin staining revealed a smaller lipid droplet size in the adipose tissues, lower lipid accumulation in the hepatocytes, and larger beta cell area in the pancreas of NAG-1 Tg mice than in those of WT mice. Immunohistochemical analysis revealed downregulated expression of cleaved caspase-3, an apoptosis marker, in the beta cells of NAG-1 Tg mice. Adiponectin and leptin mRNA levels were upregulated and downregulated in NAG-1 Tg mice, respectively. Additionally, the expression of IRS1/PI3K/AKT signaling pathway components, especially *Foxo1*, which regulates gluconeogenesis in the muscle and white adipose tissue, was downregulated in NAG-1 Tg mice. Furthermore, NAG-1 overexpression promoted the expression of *As160* in both muscles and adipocytes, and the mRNA levels of the NLRP3 pathway members were downregulated in NAG-1 Tg mice. Our findings suggest that NAG-1 expression alleviates diabetes in mice.

## Introduction

Nonsteroidal anti-inflammatory drug-activated gene 1 (NAG-1), also known as growth differentiation factor 15 (GDF15), is a member of the TGF-β superfamily. The signaling and function of NAG-1 are associated with cell and tissue homeostasis. Previous studies have reported that patients with critical illnesses or those under stress exhibit upregulated serum NAG-1 levels^1^. In contrast, the expression of serum NAG-1 is downregulated under normal physiological conditions^2^. Recent studies have indicated that NAG-1 is a major regulator of energy metabolism and appetite^3^. Additionally, NAG-1 is reported to contribute to the pathogenesis of various diseases, such as cardiovascular diseases, obesity, inflammation, cognitive impairment, and cancer^1^. NAG-1 exerts its effects, probably through mature NAG-1 and pro-NAG-1 forms, which are secreted into the extracellular matrix^4–6^. In contrast to mature NAG-1, the biological activity of pro-NAG-1 has not been extensively investigated, except that the nuclear translocation of intercellular pro-NAG-1 regulates the transcription of members of the SMAD pathways^4^.

The increasing prevalence of diabetes is a global health concern. Type 2 diabetes mellitus (T2DM) is characterized by persistent hyperglycemia. The major phenotypes of T2DM include dysregulated insulin response in the corresponding cells and insufficient insulin production in the pancreas. Furthermore, prolonged high blood glucose levels can lead to other complications, such as diabetic retinopathy, diabetic neuropathy, diabetic foot, cognitive deficits, and cardiovascular diseases^7^. Obesity and a sedentary lifestyle are the major causes of diabetes that contribute to increased morbidity and mortality^8^.

There are contradictory reports on the role of NAG-1 in diabetes. For example, in a study examining the correlation between NAG-1 and beta cell function, serum NAG-1 levels were upregulated in the prediabetes and diabetes groups. Additionally, the upregulated serum NAG-1 levels were correlated with HbA1c, glucose, insulin, baseline, and dynamic indices of insulin sensitivity^9^. These results suggest that glucose upregulates serum NAG-1 levels in patients with diabetes, indicating that serum NAG-1 could be a potential marker for identifying individuals who are at risk for developing diabetes and obesity. Conversely, NAG-1 Tg mice exhibit increased energy metabolism, decreased body weight, and improved glucose and insulin responses^10^. Moreover, the administration of recombinant NAG-1 decreases food intake and body weight, which results in decreased adiposity and improved glucose tolerance and insulin sensitivity in obese and wild-type mice^11^. Interestingly, metformin, a first-line drug for T2DM, promotes body weight loss through the upregulation of NAG-1^12^. Although the role of NAG-1 in the pathogenesis of diabetes is unclear, the expression of NAG-1, in both mature NAG-1 and pro-NAG-1 forms, may play a role in the disease process.

The correlation between obesity-induced chronic inflammation and insulin resistance has been extensively investigated. The NLR family pyrin domain containing 3 (NLRP3) inflammasome, which plays an important role in regulating inflammatory cytokines, is associated with metabolic disorders^13^. Activation of the NLRP3 inflammasome promotes the secretion of interleukin-1β (IL-1β) and interleukin-18 (IL-18), which induce inflammation. The downregulation of NLRP3 inflammasome activity in NAG-1 Tg mice may confer resistance against diet-induced obesity and improve insulin sensitivity^14^. Another study suggested that IL-1β and IL-18 are associated with obesity-associated inflammation and promote insulin resistance^15^. Thus, NLRP3 may be a novel therapeutic target to alleviate inflammasome hyperactivation in patients with diabetes.

The goal of this study was to elucidate the role of NAG-1 in insulin signaling, which is the major pathway that regulates cellular metabolic pathways, such as those involved in glucose and lipid metabolism. Mice were fed a high-fat diet (HFD) and administered a low dose of streptozotocin (STZ) to induce T2DM, and the IRS1/AKT/PI3K signaling pathway was examined. The findings of this study indicate that NAG-1 regulates the insulin pathway; thus, NAG-1 could be a biomarker for determining the efficacy of anti-diabetic agents.

## Results

### NAG-1 Tg mice exhibit decreased body weight and blood glucose level

**Figure 1:**
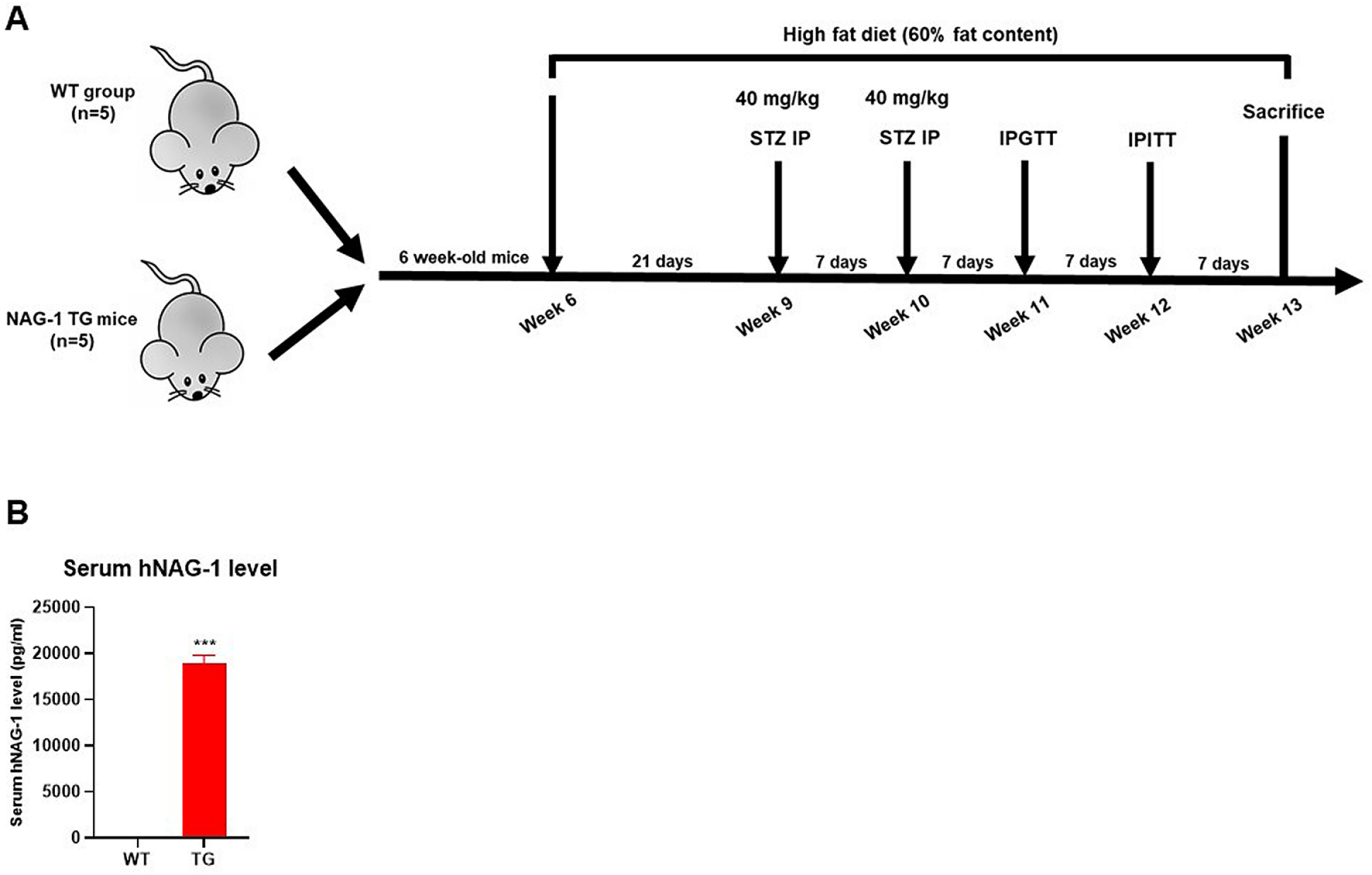
(**A**) Schematic diagram representing the timeline for experiments using the diabetes mouse model. NAG-1-overexpressing transgenic (n = 5) and wild-type (n = 5) male mice were fed with high-fat diet throughout the experiment. The mice were administered low-dose streptozotocin at weeks 9 and 10. Individual mice with blood sugar levels of > 200 mg/dL were considered to be animals with type 2 diabetes mellitus. (**B**) ELISA assay for human NAG-1 level in mouse serum. Data are presented as mean ± standard error, ***p < 0.01, compared with the wildtype group.

**Figure 2:**
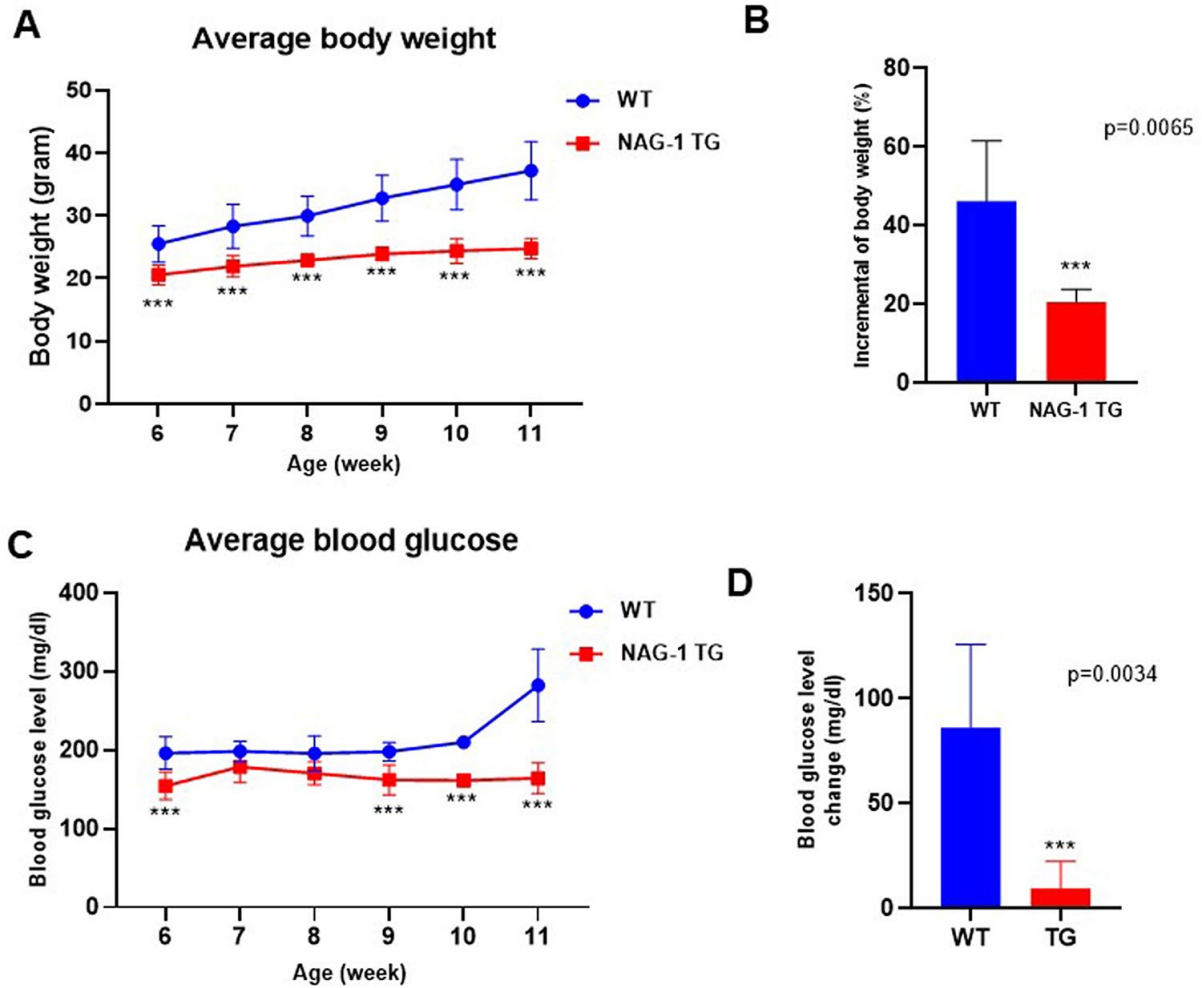
Effects of NAG-1 on body weight and glucose level in high-fat diet/streptozotocin-induced diabetes mouse model. (**A**) Body weight, (**B**) change in body weight, (**C**) blood glucose level, and (**D**) change in blood glucose level. Data are presented as mean ± standard error, ***p < 0.01, compared with the wild-type group. n = 5 for all experiments.

It has been reported that the body weight of NAG-1 Tg mice is lower than that of their sibling WT mice, in both genetically modified and HFD-induced obese animal models^10,16,17^. In this study, the effect of NAG-1 expression on STZ-treated mice was investigated (Fig. 1A). NAG-1 Tg mice express ~ 19 ng/ml of human NAG-1 in their serum, whereas no detectable human NAG-1 was found in their sibling WT mice (Fig. 1B). The body weight of NAG-1 Tg mice was significantly lower than that of WT mice (Fig. 2A), which concurred with the findings of a previous study on non-STZ-treated mice^10^. The incremental changes in body weight between weeks 6 and 11 in NAG-1 Tg mice (20.65 ± 3.27%) were markedly lower than those in WT mice (46.24 ± 15.35%) (Fig. 2B). The blood glucose levels in NAG-1 Tg mice significantly decreased, especially after week 9, which was the time point at which STZ treatment was initiated (Fig. 2C). The change in blood glucose level between the baseline and final week in WT mice (86.20 ± 39.63 mg/dL) was markedly higher than that in NAG-1 Tg mice (9.60 ± 12.85 mg/dL) (Fig. 2D). Thus, the body weight and blood glucose levels decreased in NAG-1 Tg mice, indicating that NAG-1 expression decreases blood glucose levels in the HFD/STZ-induced diabetic mouse model.

### NAG-1 Tg mice exhibit increased insulin sensitivity

**Figure 3:**
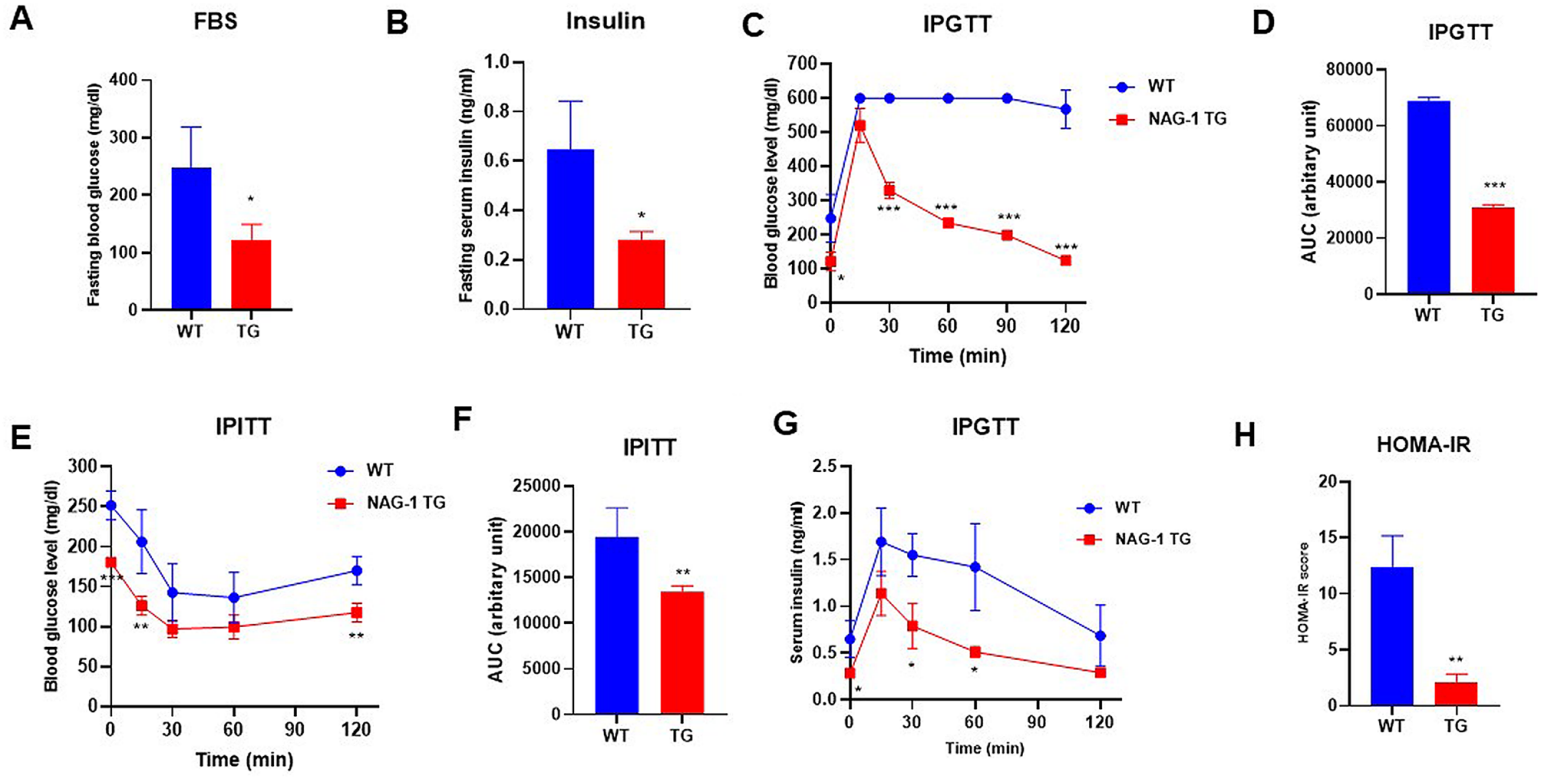
Anti-diabetic effect of NAG-1 in high-fat diet/streptozotocin-induced diabetes mouse model. Mice were allowed to fast for 18 h before the blood sample was collected. (**A**) Fasting blood glucose level and (**B**) fasting insulin level at week 11 before starting the intraperitoneal glucose tolerance test (IPGTT). (**C**) Fasting blood glucose levels at the indicated time points in the IPGTT. Mice were allowed to fast for 18 h before the administration of glucose (2 g/kg body weight). The blood glucose levels were measured at 0, 15, 30, 60, 90, and 120 min post-glucose injection. (**D**) The area under the curve of glucose. (**E**) Fasting blood glucose levels at the indicated time point in the intraperitoneal insulin tolerance test (IPITT). Mice were allowed to fast for 6 h before the intraperitoneal administration of insulin (0.75 U/kg bodyweight). The blood samples were collected to measure the serum glucose at 0, 15, 30, 60, and 120 min post-insulin injection. (**F**) The area under the curve of glucose. (**G**) Serum insulin levels in the IPGTT at the indicated time points. (**H**) Homeostatic model assessment of insulin resistance scores. Data are presented as mean ± standard error of mean. *p < 0.05, **p < 0.01, and ***p < 0.001, compared with the wild-type group (n = 3–5).

The levels of fasting blood glucose and insulin were measured, and the intraperitoneal glucose tolerance test (IPGTT) and intraperitoneal insulin tolerance test (IPITT) were performed before the mice were euthanized. The fasting blood glucose levels in NAG-1 Tg mice were significantly lower than those in WT mice (121.67 ± 27.79 mg/dL vs. 248.33 ± 70.30 mg/dL; p = 0.0216) (Fig. 3A). Additionally, the fasting insulin levels were significantly lower in NAG-1 Tg mice than in WT mice (0.28 ± 0.03 vs. 0.65 ± 0.19 ng/mL; p = 0.0341) (Fig. 3B). Glucose tolerance and insulin sensitivity were measured in both NAG-1 Tg and WT mice. WT mice exhibited higher serum glucose levels than NAG-1 Tg mice during the 120-min period after glucose administration (Fig. 3C,D). The area under the curve (AUC) of glucose in NAG-1 Tg mice was significantly lower than that in WT mice (31,037 ± 924.55 vs. 68,877.76 ± 1250.99; p < 0.001) (Fig. 3D). To further examine insulin sensitivity, IPITT was performed at different time points (Fig. 3E). The mice were intraperitoneally administered insulin, and blood samples were collected to measure glucose levels. Glucose levels (Fig. 3E) significantly decreased after insulin injection and 120 min after insulin administration in NAG-1 Tg mice. However, the glucose levels were not significantly different between the groups 30- and 60-min after insulin injection. The AUC of serum glucose (Fig. 3F) in NAG-1 Tg mice was significantly lower than that in WT mice (13,440.33 ± 645.22 vs. 19,423.00 ± 3221.49; p = 0.0344). The serum insulin levels were determined through IPGTT (Fig. 3G) significantly decreased 30 min after glucose injection. These results demonstrated that NAG-1 Tg mice were more sensitive to insulin than WT mice. Insulin resistance and insulin sensitivity were calculated using the homeostatic model assessment of insulin resistance (HOMA-IR) (Fig. 3H). The HOMA-IR score (2.14 ± 0.69) in NAG-1 Tg mice was markedly lower than that (12.33 ± 2.86) in WT mice, which indicated that NAG-1 overexpression increases insulin sensitivity in mice.

### Diabetic dyslipidemia and hepatic steatosis in the HFD/STZ-induced diabetic mouse model

**Figure 4:**
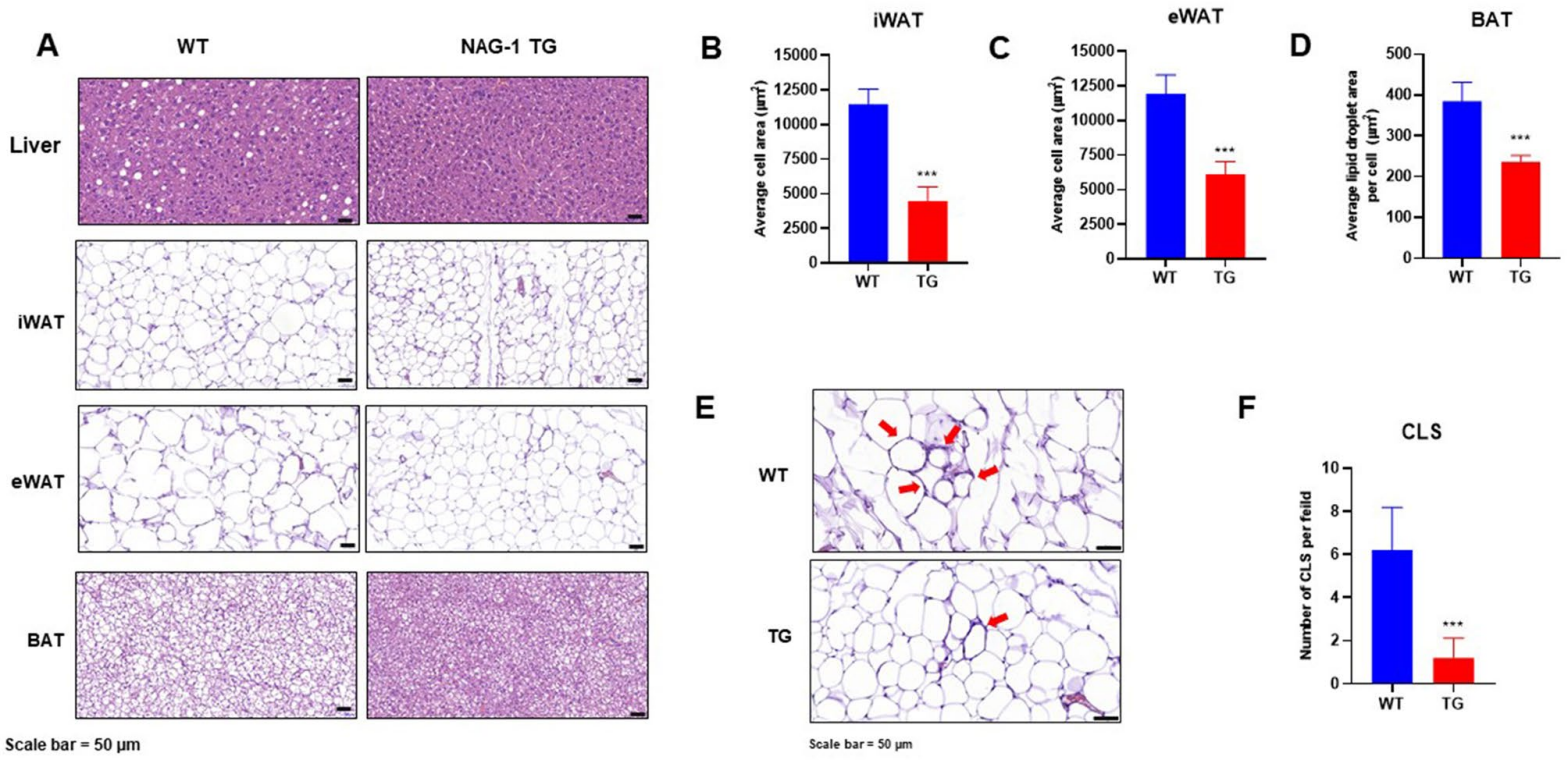
NAG-1 overexpression attenuated high-fat diet/streptozotocin-induced diabetic dyslipidemia and hepatic steatosis in mice. The tissue sections were prepared and stained with hematoxylin and eosin (H&E). (**A**) Representative image of the H&E-stained section of the liver, iWAT, eWAT, and BAT. Scale bar = 50 µm. (**B**–**D**) Average lipid droplet area in the iWAT, eWAT, and BAT. (**E**) Histological staining of crown-like structure (CLS) in the eWAT. Scale bar = 50 µm. (**F**) Number of CLS per field (10 fields per group). Data are presented as mean ± standard error of mean. *p < 0.05 and ***p < 0.001, compared with the control group.

Histological staining images of the liver, inguinal white adipose tissue (iWAT), epididymal white adipose tissue (eWAT), and brown adipose tissue (BAT) are shown in Fig. 4A. Lipid accumulation and the number of fat droplets in the hepatic sections were lower in NAG-1 Tg mice than in WT mice. This suggests that NAG-1 overexpression decreases hepatic steatosis. Furthermore, adipose tissue hypertrophy in NAG-1 Tg mice was lower than that in WT mice. Quantification of the histological data revealed that the size of lipid droplets in the iWAT, eWAT, and BAT was significantly smaller in NAG-1 Tg mice than in WT mice (p < 0.001) (Fig. 4B–D). The number of crown-like structures (CLS) in the eWAT, a histological hallmark of the pro-inflammatory process in the adipose tissue, was significantly lower in NAG-1 Tg mice than in WT mice (p < 0.001), which suggested that there were fewer inflammatory lesions in NAG-1 Tg mice (Fig. 4E,F). The number of CLS in the iWAT was not significantly different between WT and NAG-1 Tg mice (data not shown).

### Effects of NAG-1 overexpression on pancreatic islet architecture in the HFD/STZ-induced diabetic mouse model

**Figure 5:**
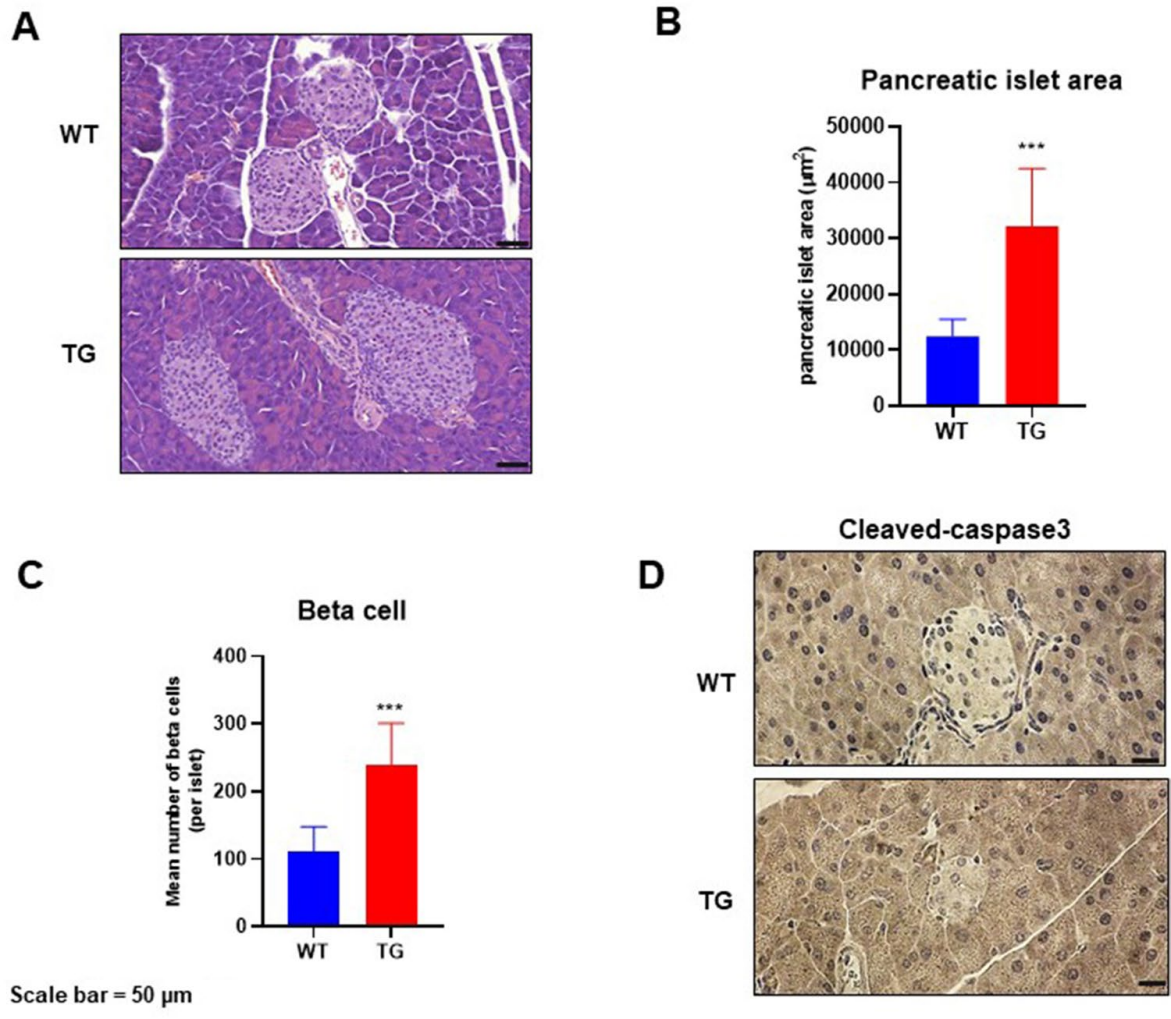
Pancreatic islet architecture. The pancreatic sections were prepared and stained with hematoxylin and eosin (H&E) or anti-cleaved caspase-3 antibody. (**A**) Representative images of H&E-stained pancreatic sections from different groups. Scale bar = 50 µm. (**B**,**C**) Pancreatic islet area and beta cell number were measured as described in the methods section. Data are shown as mean ± standard error of mean. ***p < 0.01. (**D**) Immunohistochemical analysis of cleaved caspase-3 in the pancreas islets. Scale bar = 50 µm.

T2DM development is accompanied by the loss of β cells in the pancreatic islets^18^. The effect of NAG-1 overexpression on pancreatic islets was examined using hematoxylin and eosin (H&E) staining and immunohistochemistry. H&E analysis of the pancreas (Fig. 5A–C) revealed that the islet area in NAG-1 Tg mice was significantly larger than that in WT mice (32,138 ± 10,374.47 µm^2^ vs. 12,459.39 ± 3072.69 µm^2^; p = 0.004). The mean number of beta cells per islet in NAG-1 Tg mice was significantly higher than that in WT mice (238.63 ± 61.88 cells/islet vs. 109.88 ± 37.78 cells/islet; p = 0.002). Immunohistochemical analysis revealed that the expression of cleaved caspase-3, a hallmark of apoptosis^19^, in the pancreatic islets of NAG-1 Tg mice was lower than that in the pancreatic islets of WT mice (Fig. 5D). This indicates that NAG-1 overexpression delays the development of T2DM and decreases apoptosis in pancreatic islets.

### Effect of NAG-1 overexpression on mRNA expression level of the insulin signaling pathway members

**Figure 6:**
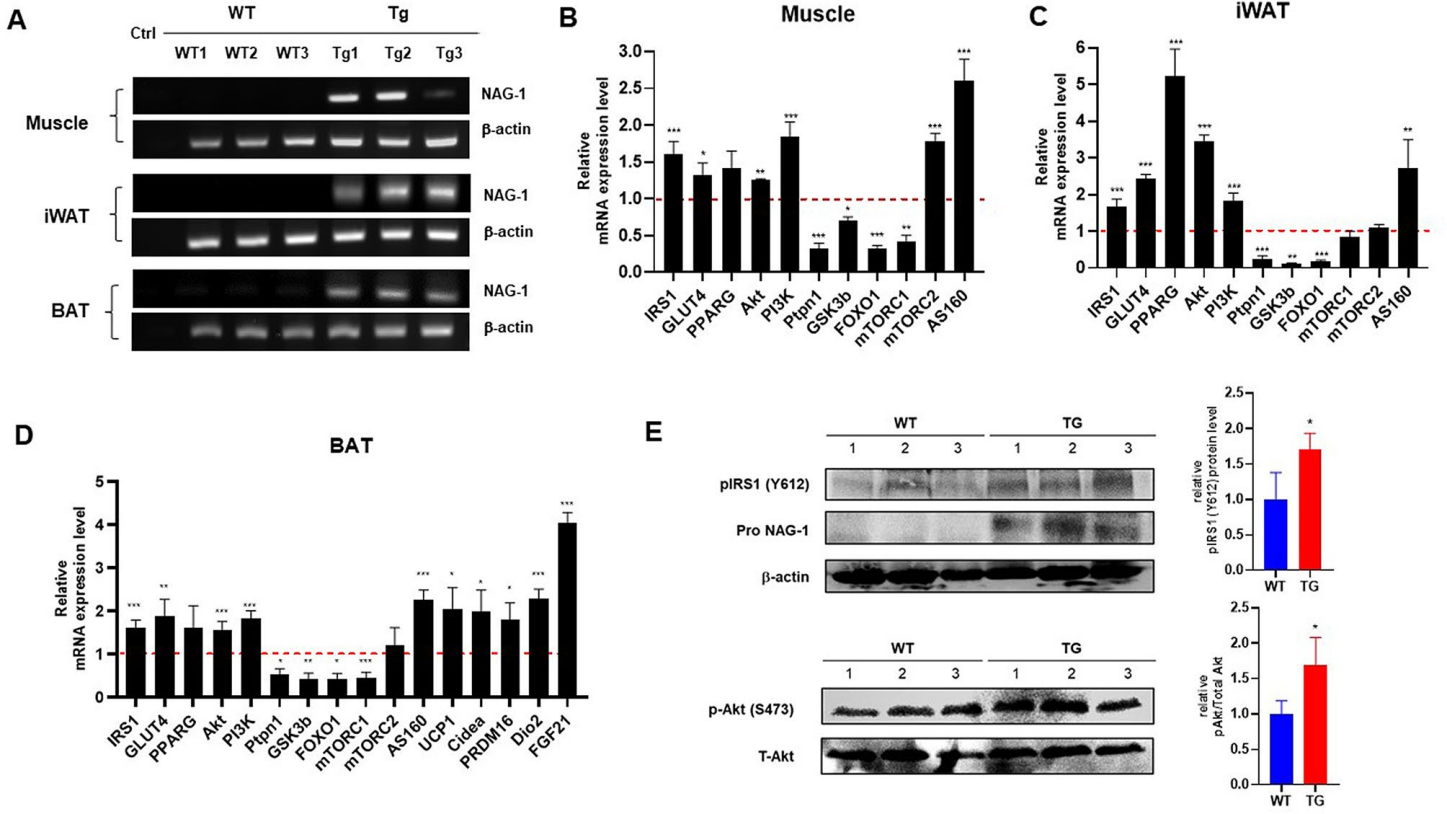
Effect of NAG-1 on the mRNA expression level of insulin signaling pathway-related factors. (**A**) RT-PCR of NAG-1 gene expression in the muscle, iWAT, and BAT. Ctrl indicates negative control. The mRNA expression levels of *Irs1*, *Glut4*, *Pparg*, *Akt*, *Pi3k*, *Ptpn1* (*Pten*), *Gsk3b*, *Foxo1*, *Mtorc1*, *Mtorc2*, and *As160* in the (**B**) muscle tissue, (**C**) inguinal white adipose tissue, and (**D**) brown adipose tissue were examined using quantitative real-time polymerase chain reaction. The thermogenesis related mRNA level of *Ucp1, Cidea, Prdm16, Dio2* and *Fgf21* were also determined in BAT. The expression levels of target genes were normalized to those of *Gapdh*. Data were analyzed using the Student’s *t*-test and are presented as mean ± standard error of mean. *p < 0.05, **p < 0.01, and ***p < 0.001, compared with the wild-type group (n = 3). (**E**) Western blot data represent the protein expression of p-Akt (Ser473), total Akt, pIRS1 (Tyr612) and pro-NAG-1 in skeleton muscle of WT diabetic mice (WT) and NAG-1 Tg diabetic mice (TG). The graph represents the relative protein expression. *p < 0.05, compared with the wildtype group. (n = 3 mice per group).

To confirm NAG-1 expression in NAG-1 Tg mice, we performed real-time polymerase chain reaction (RT-PCR) on muscle, iWAT, and BAT. The results indicated the expression of human NAG-1 in NAG-1 Tg mice, whereas no NAG-1 mRNA expression was observed in WT mice (Fig. 6A). To investigate the molecular mechanisms underlying the NAG-1-mediated regulation of blood glucose levels, a quantitative RT-PCR (qRT-PCR) analysis was performed to determine the expression levels of several genes related to the insulin signaling pathway. In NAG-1 Tg mice, the mRNA expression of *Irs1*, *Glut4*, *Akt*, *Pi3k*, and *As160* was upregulated in the muscle, iWAT, and BAT (Fig. 6B–D). Western blot data showed an increase in phospho-Akt protein expression in NAG-1 Tg mice, indicating that NAG-1 regulates the phosphorylation of Akt at Ser473. Thus, NAG-1 may improve glucose metabolism and attenuate insulin resistance via the activation of the IRS1/AKT/PI3K signaling pathway. These results were also confirmed by measuring the mRNA expression levels of the downstream effectors of the AKT pathway, including *Gsk3b*, *Foxo1*, and *Mtorc1*. The expression levels of downstream effector genes were significantly downregulated in the muscles, iWAT, and BAT of NAG-1 Tg mice. Additionally, the expression level of *Ptpn1*, a negative regulator of AKT, was downregulated in the muscles, iWAT, and BAT of NAG-1 Tg mice. Interestingly, the expression level of *Mtorc2* was significantly upregulated in the muscles but not in the iWAT and BAT. A previous study reported that *Mtorc2* in muscle tissue contributes to glucose homeostasis by positively regulating the insulin-stimulated phosphorylation of the *Akt* substrate *As160* and negatively regulating basal glycogen synthase activity^20^. In contrast, *Mtorc2* in adipose tissues controls the expression of the lipogenic transcription factor ChREBPβ, which increases de novo lipogenesis in adipose tissue and impairs hepatic insulin sensitivity^21^. The mRNA expression level of *Pparg* was significantly upregulated in the iWAT and BAT, but not in the muscles. As adipose tissue is the major mediator of PPARγ activity on insulin sensitivity, PPARγ activation in mature adipocytes induces the expression of several genes involved in the insulin signaling cascade and, consequently, enhances insulin sensitivity^22^. Furthermore, the thermogenesis related-genes including *Ucp1, Cidea, Prdm16,* and *Fgf21*, significantly increased in BAT of NAG-1 Tg mice, indicating that NAG-1 plays roles in thermogenesis (Fig. 6D). To confirming the effect of NAG-1 on insulin signaling at the post-translational level, we performed the western blot. The results indicated that NAG-1 Tg mice showed a significant positive effect on insulin signaling by enhancing phosphorylated IRS (insulin receptor substrate) at Tyr612 and its downstream Akt by increasing phosphorylation of Akt at ser473 (Fig. 6E).

### Effect of NAG-1 overexpression on the mRNA expression levels of adipocytokines

**Figure 7:**
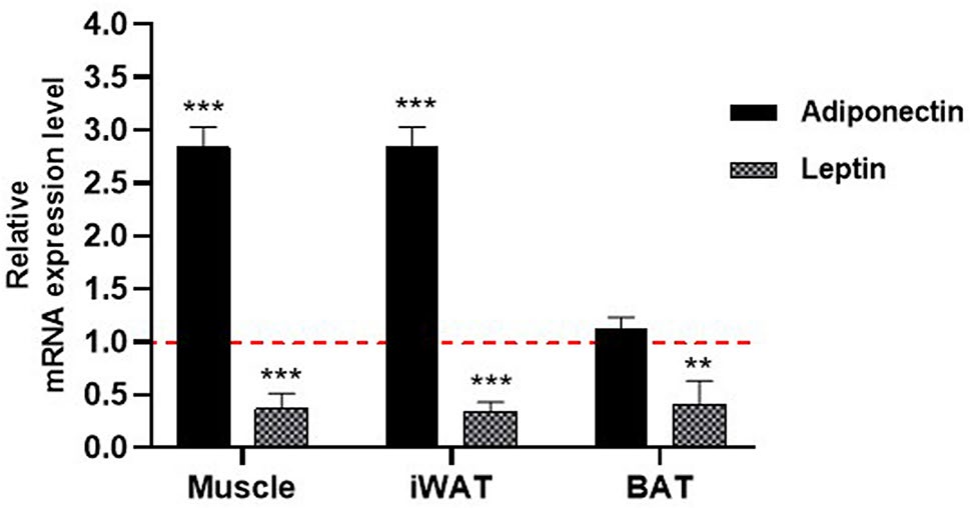
Effect of NAG-1 on the mRNA expression level of adipocytokines. Adiponectin and leptin mRNA expression levels in the muscle tissue, inguinal white adipose tissue, and brown adipose tissue were determined using quantitative real-time polymerase chain reaction. The expression levels of target genes were normalized with those of *Gapdh*. Data are presented as mean ± standard error and were analyzed using the Student’s *t*-test. *p < 0.05, **p < 0.01, and ***p < 0.001, compared with the control group (n = 3).

Adiponectin and leptin, major adipokines, are important mediators of energy homeostasis. Leptin decreases appetite, stimulates thermogenesis, enhances fatty acid oxidation, decreases glucose levels, and reduces body weight and fat mass^23^. The effect of NAG-1 overexpression on the mRNA expression levels of adiponectin and leptin was examined in NAG-1 Tg mice. The mRNA level of adiponectin was upregulated, whereas that of leptin was downregulated, in the muscles and iWAT of NAG-1 Tg mice. However, the mRNA level of adiponectin in BAT was not significantly different between WT and NAG-1 Tg mice (Fig. 7).

### Effect of NAG-1 overexpression on the mRNA expression level of NLRP3 inflammasome components

**Figure 8:**
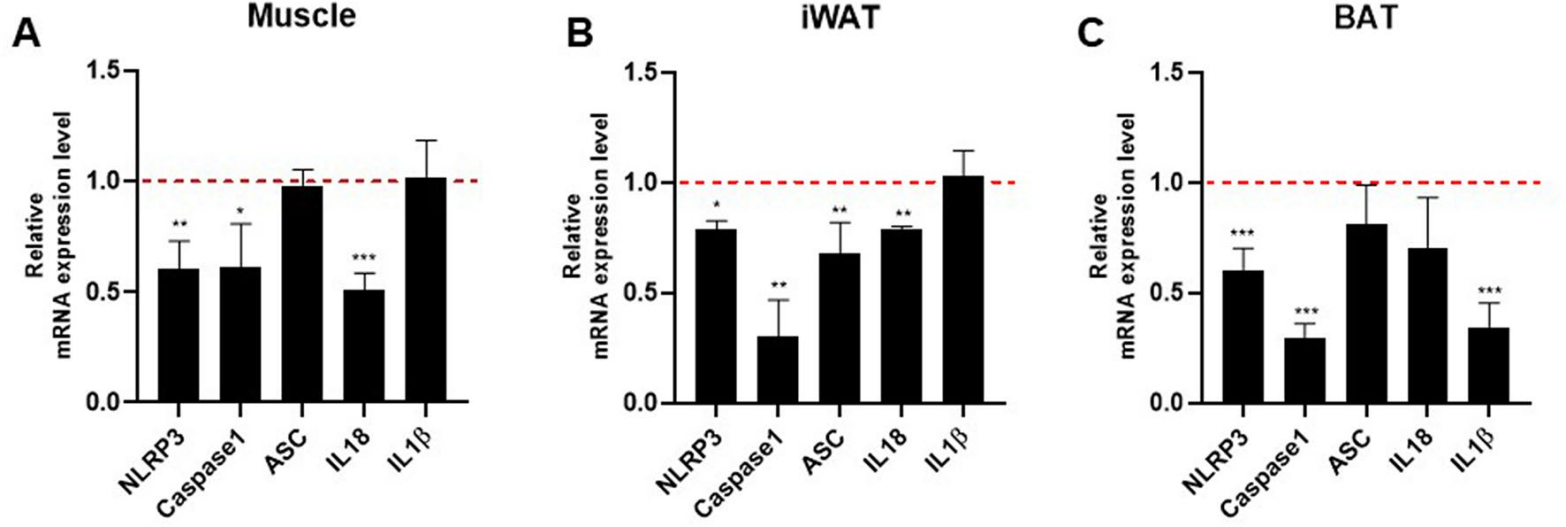
Effect of NAG-1 on the mRNA expression level of NLRP3 inflammasome components. The mRNA expression levels of *Nlrp3*, *Casp1*, *Asc*, *Il-18*, and *Il-1b* in the (**A**) muscle, (**B**) inguinal white adipose tissue, and (**C**) brown adipose tissue. The expression levels of target genes were normalized to those of *Gapdh*. Data are presented as mean ± standard error of mean and were analyzed using the Student’s *t*-test. *p < 0.05, **p < 0.01, and ***p < 0.001, compared with the control group (n = 3).

NLRP3 inflammasomes are involved in the secretion of inflammatory cytokines, which promote insulin resistance^14^. The NLRP3 inflammasome contains three main components: apoptosis-associated speck-like protein containing CARD (ASC), pro-inflammatory caspase-1, and NLRP3. Therefore, this study investigated the effects of NAG-1 overexpression on NLRP3 inflammasome component expression in a mouse model of HFD/STZ-induced diabetes. The mRNA expression levels of *Nlrp3* and *Casp1* in the muscles, iWAT, and BAT of NAG-1 Tg mice were significantly lower than those in the muscles, iWAT, and BAT of WT mice (Fig. 8A–C). Compared with those in the iWAT, muscle, and BAT of WT mice, the mRNA levels of *Asc* were significantly downregulated in the iWAT but not in the muscle and BAT of NAG-1 Tg mice. Furthermore, the mRNA levels of *Il18* were significantly downregulated in the muscles and iWAT of NAG-1 Tg mice compared with those in the muscles and iWAT of WT mice. In contrast, the mRNA levels of *Il1b* in the BAT of NAG-1 Tg mice were significantly lower than those in the BAT of WT mice. The expression levels of *Nlrp3* and *Casp1* were downregulated in the muscles, iWAT, and BAT of NAG-1 Tg mice. This indicated that the decreased NLRP3 inflammasome activity in NAG-1 Tg mice alleviates diet-induced obesity and enhances insulin sensitivity in the HFD/STZ-induced diabetic mouse model.

## Discussion

Mature NAG-1/GDF15 signaling in the brain exerts anti-obesity effects by regulating appetite^24^ and, consequently, decreasing body weight and fat mass^17^. Consistently, this anti-obesity effect is inhibited in NAG-1 knockout mice^16^. Previously, we reported that NAG-1 is a novel therapeutic target for obesity and insulin resistance as it modulates metabolic activity by promoting the expression of key thermogenic and lipolytic genes in BAT and WAT^10^. These NAG-1 transgenic mice were developed using Protamine-Cre mice, indicating that NAG-1 expression is not toxic to mice and that all tissues express NAG-1^17^. As NAG-1 can decrease body weight, it has potential applications in the prevention and treatment of obesity and hyperglycemia, which is one of the main chronic symptoms of diabetes.

The molecular mechanisms of action of NAG-1 in diabetes have not been elucidated. However, recent studies have suggested that treatment with NAG-1/GDF15 inhibits diabetes in 53% of non-obese diabetic mice^25^. Further evidence of the anti-diabetic role of NAG-1 was obtained from the use of metformin, an anti-diabetic drug; treatment with metformin consistently induces weight loss in individuals with or without T2DM. Additionally, metformin has been reported to upregulate the expression of NAG-1/GDF15^12^, which indicates that the mechanism of action of metformin involves the induction of NAG-1 expression. However, the serum levels of NAG-1/GDF15 in patients with T2DM are higher than those in non-diabetic individuals^26^. Additionally, the serum levels of NAG-1/GDF15 are positively correlated with blood glucose concentrations^27^. The mechanism underlying the upregulation of serum NAG-1/GDF15 levels in patients with diabetes has not been elucidated. One potential explanation is the presence of several forms of NAG-1 in the serum. NAG-1 is synthesized in the cytoplasm and secreted in at least two different forms of NAG-1 (pro-form and mature forms). However, the molecular function of pro-NAG-1 in the serum has not been determined. The role of pro-NAG-1 in diabetes development must be examined; thus, the ratio of the mature form of NAG-1 to the pro-form of NAG-1 could be important in determining the progression of diabetes in patients. Furthermore, intercellular NAG-1 may also play a role in adipose tissue, which expresses NAG-1. Previously, we reported that pro-NAG-1 decreases SMAD activity in the nucleus through transcriptional regulation^4^, which possibly regulates several other mechanisms of transcriptional regulation, including micro RNA and long non-coding RNA expression. The identification of pro-NAG-1 function can aid in determining the role of NAG-1 in the development of diabetes.

To elucidate the mechanism involved in NAG-1/GDF15-mediated enhanced insulin sensitivity, this study investigated the insulin signaling pathway in NAG-1 Tg mouse tissues using the HFD/STZ-induced diabetic mouse model. In addition to the adipose tissue, the skeletal muscle was considered as a target tissue^28^ in which NAG-1/GDF15 was expressed (Fig. 6E). Glucose uptake by this tissue accounts for the majority of glucose disposal during the postprandial period, as well as after the challenge with exogenous insulin. The expression of GLUT4 was upregulated in the skeletal muscle of NAG-1/GDF15 Tg mice (Fig. 6B–D), supporting the hypothesis that NAG-1/GDF15 expression in the skeletal muscle may increase systemic insulin sensitivity. The upregulated expression of *Glut4* is accompanied by the induction of the expression of *As160*, which is required for the insulin-stimulated translocation of glucose transporter to the plasma membrane. Indeed, the expression of *Glut4* was also upregulated in iWAT and BAT (Fig. 6).

In obesity and diabetes, PI3K/AKT is the major insulin pathway involved in the physiological functions of various organs. The PI3K/AKT signaling pathway promotes lipid biosynthesis and inhibits lipolysis in adipose tissue. *Foxo1*, the substrate of PI3K/AKT, regulates lipolysis by modulating the expression of adipose triglyceride lipase^29^. Additionally, modulation of the PI3K/AKT signaling pathway and downstream effectors is a potential therapeutic strategy for obesity and T2DM. However, the complex mechanism of the PI3K/AKT pathway must be elucidated in future studies. This study demonstrated that the metabolic functions of the PI3K/AKT signaling pathway in the muscle and adipose tissue are critical for metabolism and that NAG-1 is a positive regulator of this pathway.

mTOR protein modulates insulin signaling by regulating several downstream components^30^. mTORC1, a downstream effector of AKT, is involved in the conversion of BAT to WAT. Previous studies have reported that mTORC1 activation in adipose tissue promotes the accumulation of lipids in BAT through the downregulation of brown adipocyte markers and upregulation of WAT markers. In skeletal muscle, *Mtorc1* regulates muscle mass by regulating protein synthesis and degradation^31^. The overactivation of mTORC1 in the muscle of obese and high-fat-fed rodents is associated with increased inhibitory phosphorylation of insulin receptor substrate-1 and impaired AKT activation, which leads to impaired insulin signaling, decreased glucose uptake by the muscle, and systemic insulin resistance^32^. The findings of this study indicate that mTORC1 is downregulated in BAT but not in WAT, suggesting decreased conversion of BAT into WAT in NAG-1 Tg mice. Hence, this may be associated with the low body weight and small size of the droplets in NAG-1 Tg mice (Figs. 2 and 4). Moreover, *Mtorc1* was also downregulated in the muscles, indicating decreased insulin resistance in NAG-1 Tg mice. mTORC2, a component of the mTOR complex, promotes AKT signaling via phosphorylation^33^. The knockout of mTORC2 in the muscle leads to the impairment of insulin signaling through GLUT4 translocation^20^. Additionally, the knockout of *Mtorc2* in adipose tissues suppresses insulin-induced lipolysis, which increases the levels of circulating fatty acids and glycerol^34^. The findings of this study indicate that the expression of *Mtorc2* changes in the muscle but not in the adipose tissue of NAG-1 Tg mice. This suggests that the induction of *Mtorc2* expression in the muscles of NAG-1 Tg mice is associated with the upregulation of *As160*, which leads to the translocation of GLUT4 to the plasma membrane. Interestingly, NAG-1 regulated the expression of leptin and adiponectin in this study. Studies on animal models have revealed that leptin and adiponectin play a critical role in the prevention and control of T2DM by promoting beta cell function and survival, improving insulin sensitivity, and regulating glucose metabolism^23,35,36^. Leptin and adiponectin regulate blood glucose via several mechanisms. In this study, NAG-1 overexpression downregulated the expression of leptin and upregulated that of adiponectin (Fig. 7). Previous studies have reported that increased leptin levels are associated with insulin resistance and T2DM development. Additionally, increased leptin concentrations are associated with increased cardiovascular risk in patients with T2DM^37^. In this study, NAG-1 Tg mice exhibited downregulated expression of leptin in the WAT, BAT, and muscle and decreased insulin resistance compared with WT mice. Adiponectin also contributes to improving insulin sensitivity by locally increasing GLUT4-mediated glucose uptake. However, adiponectin also inhibits BAT activation and thermogenesis in mice by suppressing *Ucp1* expression, lipolysis, and brown adipocyte recruitment^38^. This demonstrates that increased adiponectin levels are sometimes associated with metabolic dysfunction in BAT^39^. These data are consistent with the improved insulin sensitivity and increased thermogenesis in the BAT of NAG-1 Tg mice (Fig. 6D). These results are also consistent with those of our previous study, which suggested that NAG-1 mice exhibit upregulated expression of thermogenesis-related genes in the BAT^10^.

The NLRP3 inflammasome is the most fully characterized inflammasome and belongs to the nucleotide-binding oligomerization domain-like receptor (NLR) family of pattern recognition receptors, which are found in the cytosol, detect pathogen invasion, and initiate immune responses^40^. The activation of this inflammasome leads to the promotion of the secretion of the pro-inflammatory cytokines IL-1β and IL-18, which play roles in T2DM progression^41^. Several studies have reported that the NLRP3 inflammasome and its components are crucial mechanisms that induce metabolic inflammation and insulin resistance^42^. In this study, the results showed a decrease in *Nlrp3* and *Casp1* mRNA expression in the muscle, iWAT, and BAT, followed by the reduction in *Il-1β* and *Il-18* mRNA expression in different tissues of NAG-1 Tg diabetic mice compared with WT mice. Similarly, we reported that NAG-1 Tg mice fed CHO, low-fat, or-high-fat diet, exhibit a lower expression of *Nlrp3* mRNA and significantly lower expression of *Casp1*, *Asc*, *Il-1β*, and *Il-18* mRNA in the WAT than WT mice^10^. This suggests that NAG-1 Tg mice have a negative correlation with NLRP3 inflammasomes, which play important roles in protection against insulin resistance.

**Figure 9:**
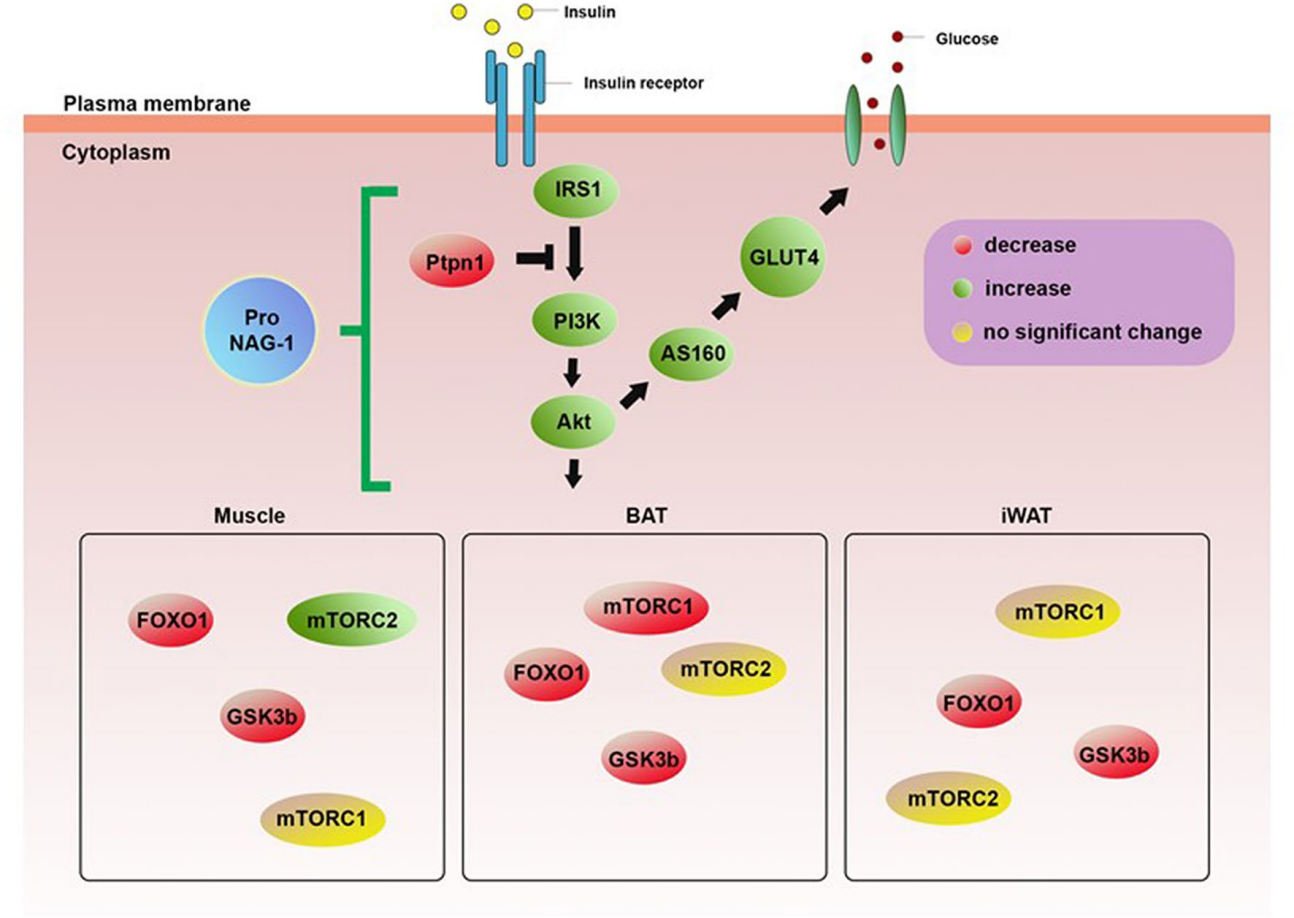
Schematic diagram representing the effect of NAG-1 on the insulin signaling pathway. Overexpression of pro-NAG-1 increases expression levels of IRS1/PI3K/AKT, resulting in anti-diabetic activity. Green and red colors represent upregulation and downregulation, respectively, whereas the yellow color indicates no significantly different expression.

In summary, the findings of this study demonstrate that NAG-1 may inhibit diabetic development in an HFD/STZ-induced diabetic mouse model. Based on the findings of this study, we conclude that NAG-1 upregulates insulin signaling and, consequently, downregulates the expression of *Gsk3β*, *mTorc1,* and *Foxo1*, which may contribute to the prevention of HFD/STZ-induced diabetic mice (Fig. 9). Histological analysis revealed that NAG-1 mitigates the adverse effects of diabetes on liver and adipose tissues. NAG-1 overexpression exerts anti-diabetic activity by increasing the pancreatic islet area and beta cell population in the pancreas. These results also highlight the potential role of NAG-1 in the development of novel preventive approaches to manage insulin resistance.

## Methods

### Animals

Six-week-old male C57BL/6 mice (high-fat-induced WT and NAG-1 Tg mice) were used in this study. The animals were housed in a temperature-controlled (21 ± 2 °C) room with a 12-h dark/light cycle. The mice had free access to food and water. All mice were maintained on an HFD (60% from fat, D12492, Research Diets, Inc., New Brunswick, NJ, USA) throughout the experimental period. All procedures used in the animal experiments complied with the standards set out in the Guidelines for the Care and Use of Laboratory Animals of Seoul National University and were approved by the Institutional Animal Care and Use Committee of Seoul National University (SNU-170417-23-2). All animals were maintained and used in accordance with ARRIVE guidelines.

### Experimental design

NAG-1 transgenic mice were previously generated using Protamine-Cre mice^17^, indicating that NAG-1 is expressed in all tissues. Six-week-old male NAG-1 transgenic mice and their NAG-1-negative sibling mice were grouped into two groups: WT mice and NAG-1 Tg mice (n = 5 for each group). All mice were maintained on HFD, and the mice were intraperitoneally injected with low-dose STZ (40 mg/kg body weight; #13104, Cayman Chemical, MI, USA) after 8 h of fasting at week 9. After injection, the mice were maintained on an HFD (Fig. 1). At week 10, the mice were administered low-dose STZ (40 mg/kg body weight, IP) for a second time to complete the induction of diabetes. The levels of fasting blood sugar (FBS) were analyzed to confirm the presence of diabetes in the mice at week 11. Enzyme-linked immunosorbent assay (ELISA) Blood was collected from a mouse (ELISA) tissues. Six-week-old male NAG-1 transgenicblood was then incubated in a serum separator tube (SST, BD Microtainer, Franklin Lakes, NJ, USA) for 30 min at room temperature. After incubation, SST was centrifuged at 15,000×*g* for 90 s to obtain the serum. These serums were used to determine the human NAG-1 protein levels in each mouse using Human GDF-15 Quantikine ELISA Kit (#DGD150, R&D Systems Inc., Minneapolis, MN, USA).

### Measurements of body weight and fasting blood sugar/insulin levels

Bodyweight and FBS levels were initially measured at week 6 and monitored every week thereafter until the end of the experimental period. FBS levels were measured using fresh blood samples collected from the mouse tail vein with Accu-Chek Performa (Roche Diabetes Care, Indianapolis, IN, USA). Fasting serum insulin levels were determined using an Ultra-Sensitive Mouse Insulin ELISA Kit (#90080, Crystal Chem, IL, USA), following the manufacturer’s instructions.

### Intraperitoneal glucose tolerance test (IPGTT)

The mice were allowed to fast for 18 h and subjected to IPGTT at week 11. Mice were intraperitoneally injected with fresh glucose solution (2 g/kg body weight, A2494001, Gibco, Gaithersburg, MD, USA), and the blood glucose level was measured at 0 (baseline), 15, 30, 60, 90, and 120 min post-glucose injection using Accu-Chek Performa. Serum insulin levels were also determined at 15, 30, 60, and 120 min post-injection using the Ultra-Sensitive Mouse Insulin ELISA Kit. The AUC value of blood glucose was calculated using GraphPad Prism 8 software (GraphPad Prism Software Inc., San Diego, CA, USA). Insulin resistance was calculated using the following formula: HOMA-IR was calculated as [fasting insulin (mU/mL) × fasting glucose (mg/dl)]/405.

### Intraperitoneal insulin tolerance test (IPITT)

At week 12, the mice were allowed to fast for 6 h before the experiment. Serum glucose levels were measured using Accu-Chek Performa at the baseline (0 min) and after the intraperitoneal injection of insulin (0.75 U/kg body weight) (I0516, Sigma-Aldrich, St. Louis, MO, USA). Blood glucose levels were determined at 15, 30, 60, and 120 min post-injection. The AUC value of blood glucose was calculated using GraphPad Prism software.

### Histological analysis

The tissues were fixed in 10% neutral formalin, embedded in paraffin, sectioned (thickness: 5 µm), and stained with H&E. Images of the tissues were captured using Panoramic SCAN (3DHISTECH, Budapest, Hungary), and the pancreatic islet area and the number of beta cells were measured using the CaseViewer program (3DHISTECH, version 2.2). The iWAT, eWAT, and BAT adipocyte areas were measured using the adipocyte tool plugin in ImageJ software 1.52a (NIH, Bethesda, MD, USA).

### Immunohistochemistry

The tissue blocks were fixed in 10% neutral formalin, embedded in paraffin, and sectioned into 5-μm-thick sections. The sections were incubated in citrate buffer for 1 min in a microwave (Immunobioscience, Mukilteo, WA, USA) to retrieve the antigens. Endogenous peroxidase activity was blocked using 0.3% hydrogen peroxide (Sigma-Aldrich, St. Louis, MO, USA) in phosphate-buffered saline for 1 h at room temperature. Immunoreactivity was detected using an ultra-sensitive ABC staining kit (Thermo Fisher, IL, USA), following the manufacturer’s instructions. The sections were incubated with rabbit polyclonal anti-cleaved caspase-3 (#9661S, Cell Signaling Technology, Beverly, MA, USA) antibody at 4 °C overnight. The negative control samples were incubated with secondary antibodies, but not with the primary antibody. The sections were incubated with 3,3′-diaminobenzidine tetrahydrochloride (DAB) substrate (ImmPACT DAB kit, Vector Laboratory, Burlingame, CA, USA) at room temperature for 45 s and counterstained with hematoxylin (Vector Laboratory, Burlingame, CA, USA) for 30 s. Coverslip-mounted sections were observed using a Panoramic SCAN slide scanner.

### RT-PCR

Total RNA was extracted from tissues using the RNeasy Mini Kit (QIAGEN, Hilden, Germany), following the manufacturer’s instructions. RNA (1 µg) was reverse-transcribed using a Verso cDNA Synthesis Kit (Thermo Fisher, IL, USA). DNA was amplified by PCR using a MiniAmp Plus Thermal Cycler (A37835, Applied Biosystems, Marsiling Industrial Estate, Singapore) with GoTaq Green PCR Master Mix (Promega, Madison, WI, USA). The primers used were human *NAG-1* (F: 5′-CTCCAGATTCCGAGAGTTGC-3′ and R: 5′-AGAGATACGCAGGTGCAGGT-3′) and mouse β-*actin* (F: 5′-GGCTGTATTCCCCTCCATCG-3′ and R: 5′-CCAGTTGGTAACAATGCCATGT-3′). The thermal cycling conditions were as follows: initial denaturation at 94 °C for 2 min, followed by 40 cycles of 94 °C for 30 s, 52 °C for 30 s, and 72 °C for 1 min, and a final extension at 72 °C for 5 min. The products were electrophoresed on a 1.5% agarose gel and visualized under UV light using an Alliance Q9 Mini (Cambridge, UK).

### qRT-PCR

Total RNA was extracted from tissues using the RNeasy Mini Kit (QIAGEN, Hilden, Germany), following the manufacturer’s instructions. RNA (1 μg) was reverse-transcribed into cDNA using a first-strand cDNA synthesis kit (K1612, Thermo Fisher, IL, USA) in a MiniAmp Plus Thermal Cycler. The primers used for qRT-PCR analysis are listed in Table 1. The relative level of each RNA was measured using qRT-PCR with SYBR Green reagents (PowerUp SYBR Green Master Mix, A25741, Applied Biosystems, Thermo Scientific) in a QuantStudio 1 real-time PCR system (Applied Biosystems, Marsiling Industrial Estate, Singapore). The expression level of the target gene was normalized to that of *Gapdh* (housekeeping gene). Relative gene expression was calculated using the comparative Ct (2^−ΔΔCt^) method.

**Table 1:** PCR primers used in this study.

	Forward primer (5′→3′)	Reverse primer (5′→3′)
Adiponectin	ATCTGGAGGTGGGAGACCAA	GGGCTATGGGTAGTTGCAGT
Leptin	AGGTAGGGATGGGTAGAGCC	GTGTGCTGCTTGGGAGTTTC
GLUT4	GGTGTGGTCAATACGGTCTTCAC	AGCAGAGCCACGGTCATCAAGA
PI3K	CAAACCACCCAAGCCCACTACT	CCATCAGCAGTGTCTCGGAGTT
Ptpn1	GCGCTTCTCCTACCTGGCTGTCAT	ACGTGCTCGGGTGGAAGGTCTA
IRS-1	TGTCACCCAGTGGTAGTTGCTC	CTCTCAACAGGAGGTTTGGCATG
Akt	GGACTACTTGCACTCCGAGAAG	CATAGTGGCACCGTCCTTGATC
GSK3β	CATAGTGGCACCGTCCTTGATC	CCAACTGATCCACACCACTGTC
PPARγ	GTACTGTCGGTTTCAGAAGTGCC	ATCTCCGCCAACAGCTTCTCCT
FOXO1	CTACGAGTGGATGGTGAAGAGC	CCAGTTCCTTCATTCTGCACTCG
AS160	GCCAACAGTCTTGCCTCAGAGA	CGTCTTCGGAACTGTGGAGAGT
RPTOR	CTTCCTATCCGTCTTGGCAGAC	CTCCAGACAGATGGCAATCAGG
RICTOR	CAGTGTGAGGTCCTTTCCATCC	GCCATAGATGCTTGCGACTGTG
UCP1	TTTTGTTCTTGCACTCACGCC	CCCATGGTGGGTTGCACTTC
PRDM16	CCACCAGCGAGGACTTCAC	GGAGGACTCTCGTAGCTCGAA
Cidea	CAGTGATTTAAGAGACGCGG	TCTGCAATCCCATGAATGTC
Dio2	ATGCTGACCTCAGAAGGGCT	ACACTGGAATTGGGAGCATC
FGF21	ATCAGGGAGGATGGAACAGTGG	AGCTCCATCTGGCTGTTGGCAA
NLRP3	TGCTCTTCACTGCTATCAAGCCCT	ACAAGCCTTTGCTCCAGACCCTAT
IL-18	TGGTTCCATGCTTTCTGGACTCCT	TTCCTGGGCCAAGAGGAAGTGATT
IL-1β	TGGACCTTCCAGGATGAGGACA	GTTCATCTCGGAGCCTGTAGTG
ASC	CTGCTCAGAGTACAGCCAGAAC	CTGTCCTTCAGTCAGCACACTG
Caspase-1	GGCACATTTCCAGGACTGACTG	GCAAGACGTGTACGAGTGGTTG
GAPDH	CATCACTGCCACCCAGAAGACTG	CATCACTGCCACCCAGAAGACTG

### Immunoblotting analysis

The tissue was washed twice in ice-cold PBS and homogenized using lysis buffer containing proteinase inhibitors. The samples were sonicated and centrifuged at 14,000×*g* for 20 min. Protein concentrations in the supernatants were determined using a Pierce BCA Protein Assay Kit (Thermo Fisher, IL, USA). After the proteins were quantified, western blotting was performed. Briefly, 60 µg of protein was separated using 9% sodium dodecyl sulfate–polyacrylamide gel electrophoresis and transferred to a nitrocellulose membrane. The blotted membrane was blocked with 5% BSA for 1 h at room temperature and incubated overnight with specific antibodies at 4 °C. The primary antibodies used (at a dilution of 1:1000) included rabbit anti-p-Akt Ser473 (#9271S, Cell Signaling, MA, USA), rabbit anti-Akt (#9272S, Cell Signaling, MA, USA), rabbit anti-p-IRS1 (Tyr612) (#44-816G, Invitrogen, MA USA), in-house rabbit anti-pro-NAG-1 (corresponding human NAG-1 amino acid peptide 43–63), anti-alpha tubulin (TU-02, #sc8035, Santa Cruz) and anti-β-actin (2A3, #sc517582, Santa Cruz). After incubation with goat anti-rabbit HRP-conjugated IgG secondary antibody (1:5000 dilution, #31460, Thermo Fisher, IL, USA) or goat anti-mouse IgG (H + L) secondary antibody (1:5000 dilution, #626520, Thermo Fisher, IL, USA), in 5% skim milk for 1 h at RT, the blotted membranes were visualized using Alliance Q9 mini (Cambridge, UK) and quantified using ImageJ software 1.52a.

### Statistical analysis

Data processing and analyses were performed using GraphPad Prism version 8.0.1. All experimental data are shown as the mean ± standard error of the mean for parametric data. The means between groups were analyzed using the Student’s *t*-test. The data from the NAG-1 Tg mice were compared with those of the controls (WT mice). Differences were considered significant at *p < 0.05, **p < 0.01, or ***p < 0.001.

## Supplementary Information

Supplementary Information.

## Data Availability

All raw and processed data for this study are provided as a supplementary file.
