# Supplementary material for: The anti-diabetic effects of NAG-1/GDF15 on HFD/STZ-induced mice
Source: Sci Rep. 2021 Jul 22;11:15027. doi: 10.1038/s41598-021-94581-y (PMC8298384; doi:10.1038/s41598-021-94581-y)
Supplement: Supplementary file 1 — Supplementary Information. [file 41598_2021_94581_MOESM1_ESM.pdf]

Supplementary information

The anti-diabetic effects of NAG-1/GDF15 on HFD/STZ-induced mice

Pattawika Lertpatipanpong[1], Jaehak Lee[1], Ilju Kim[1], Thomas Eling[2], Seung Yeon Oh[3], Je Kyung Seong[3,4], and Seung Joon Baek[1,*]

[1]Laboratory of Signal Transduction, College of Veterinary Medicine and Research Institute for Veterinary Science, Seoul National University, Seoul 08826, Korea

[2]National Institute of Environmental Health Science, 111 TW Alexander Dr. Research Triangle Park, NC 27709, USA

[3]Laboratory of Developmental Biology and Genomics, BK21 Plus Program for Advanced Veterinary Science, Research Institute for Veterinary Science, College of Veterinary Medicine, and Korea Mouse Phenotyping Center, Seoul National University, Seoul 08826, Korea.

[4]Interdisciplinary Program for Bioinformatics, Seoul National University, Seoul 08826, Korea

*Correspondence to: *baeksj@snu.ac.kr (S.J.Baek)*

Supplementary Figure 1. The expression of pro NAG-1 protein in muscle and pancreas.

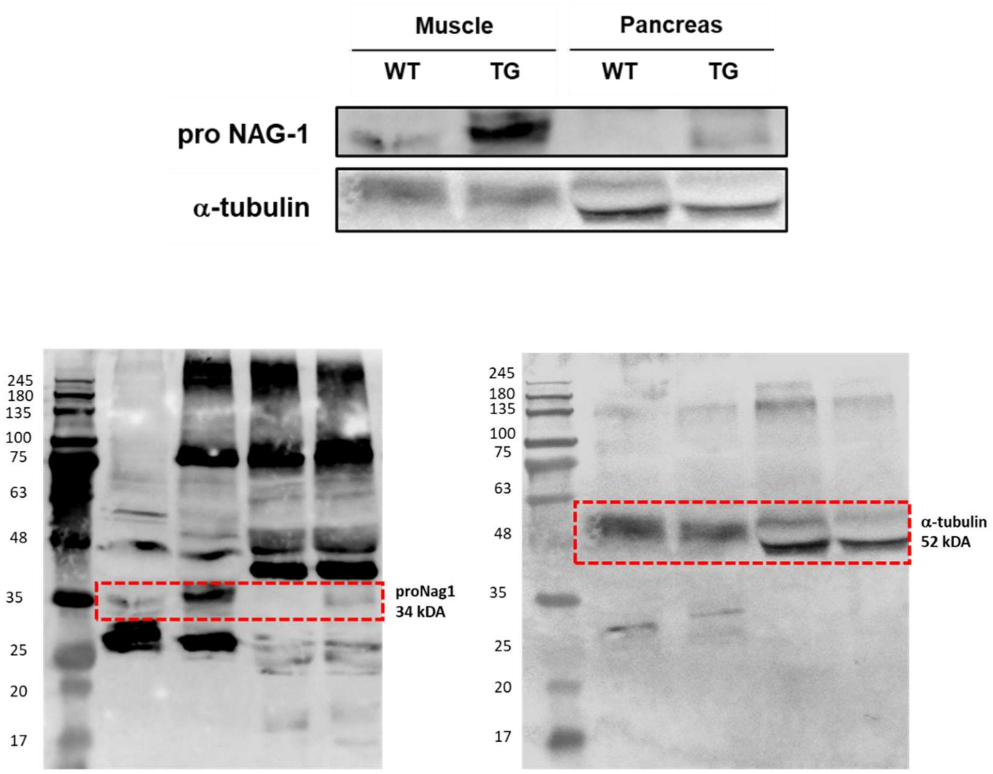

Supplementary Figure 2. Morphologies of pancreas Islets.

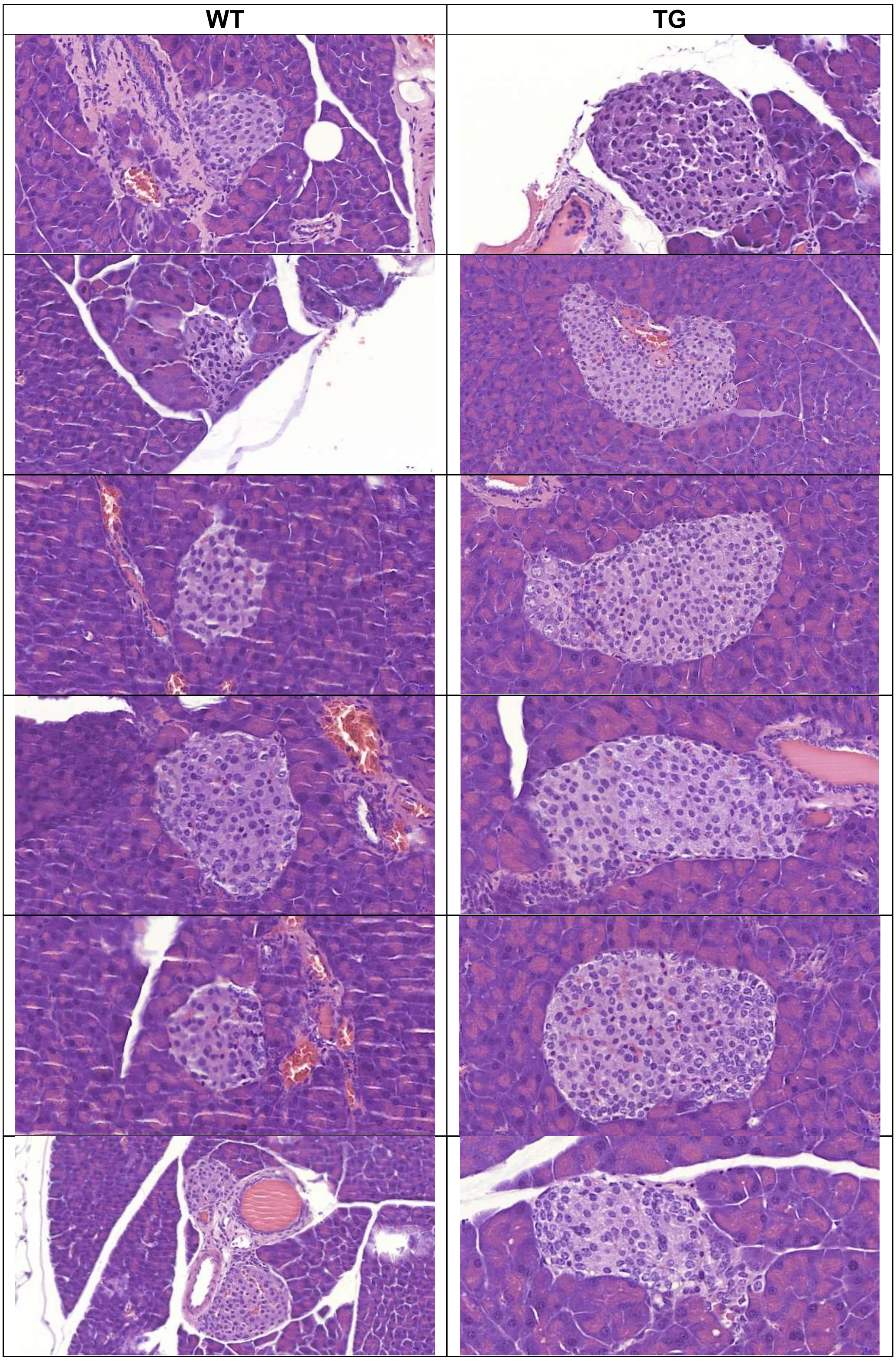

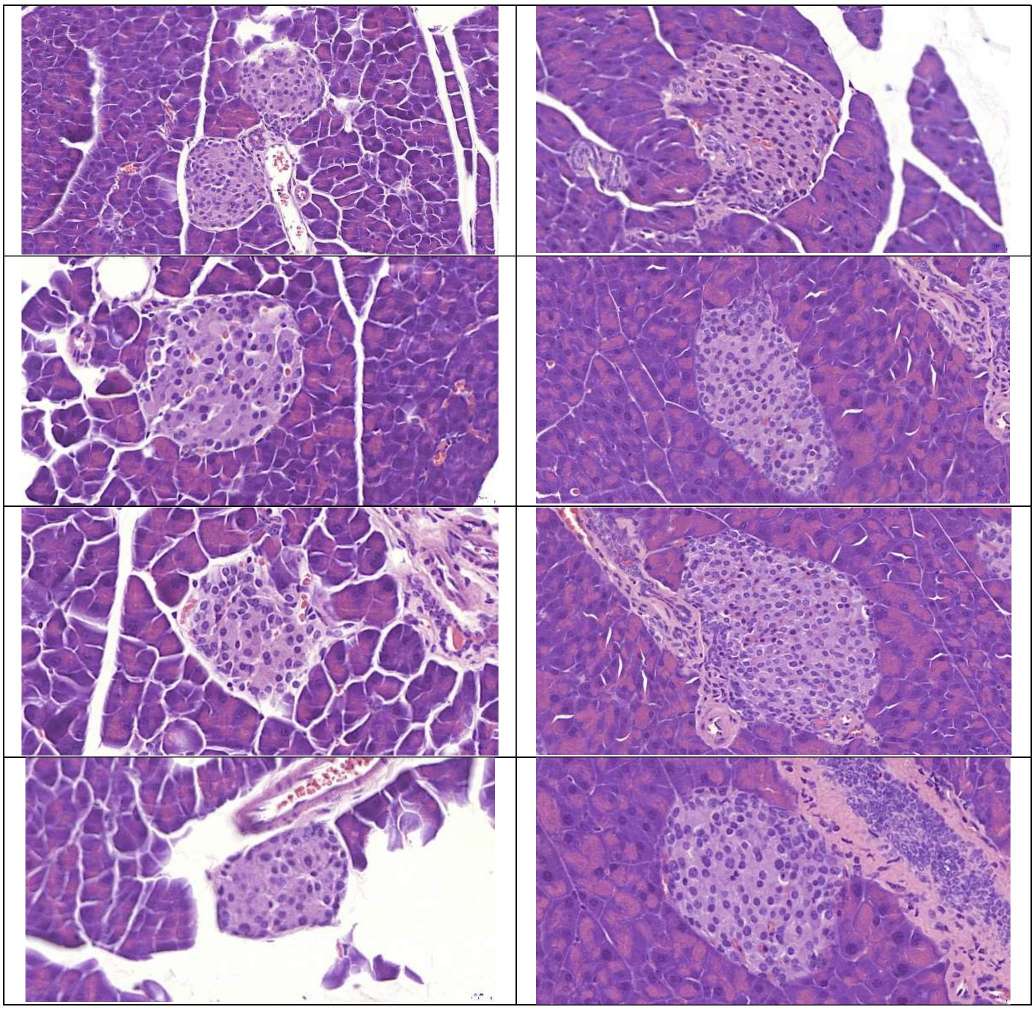

Supplementary Figure 3. Crown-like structure in adipose tissues.

WT	TG

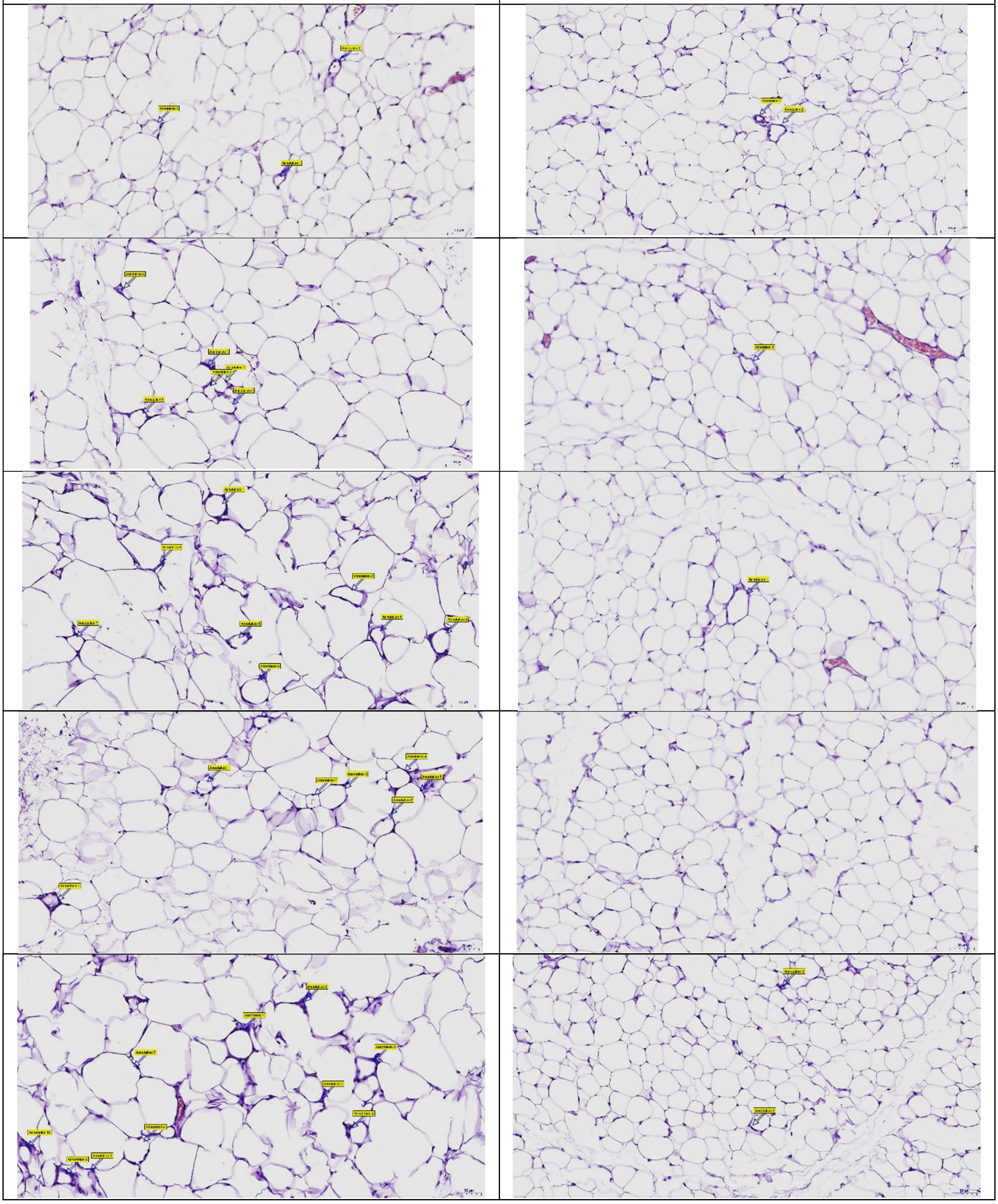

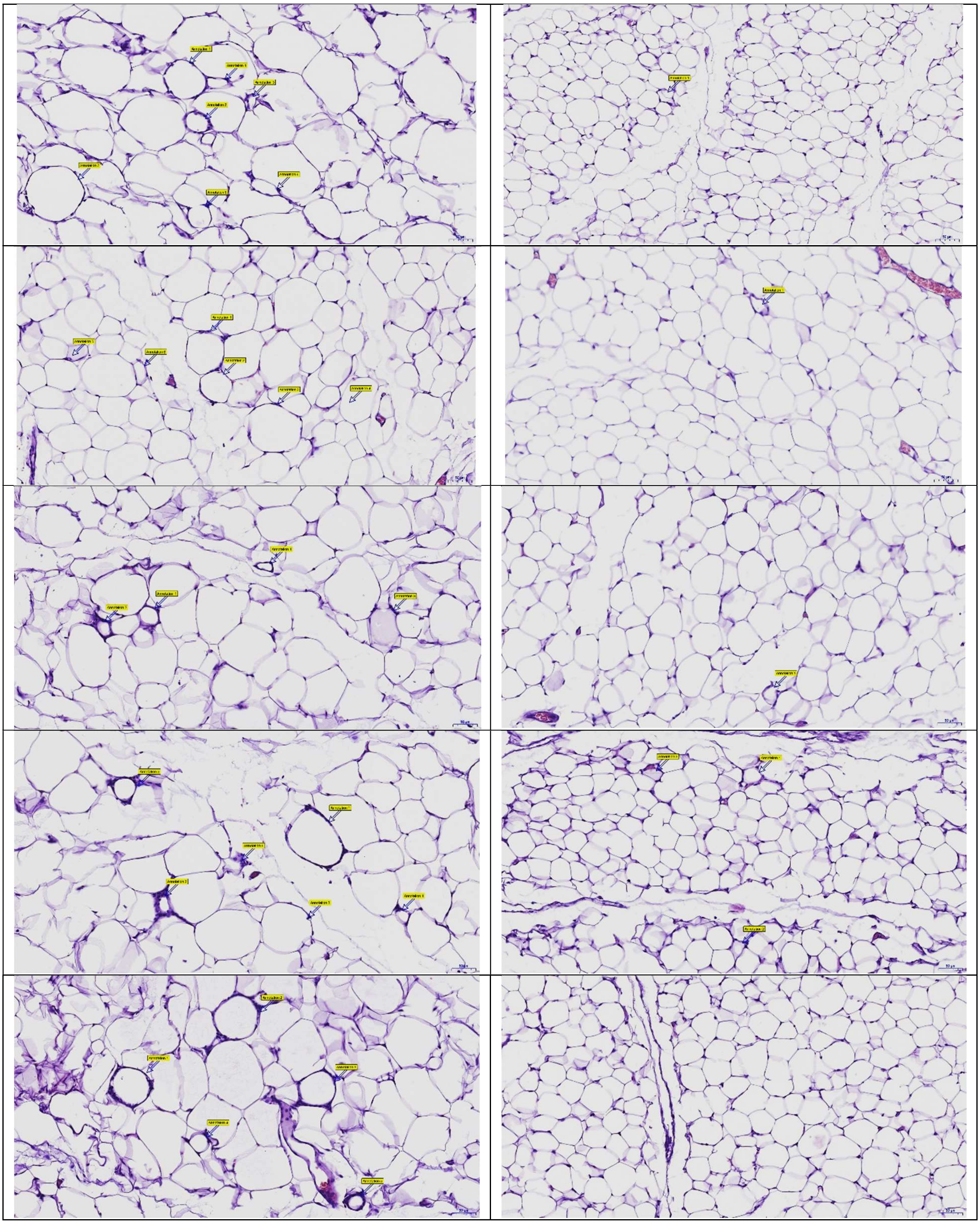

Supplementary Figure 4. The expression of human NAG-1 and β-actin in muscle, iWAT and BAT using RT-PCR. (*hNag1* = 196 bp, *β-actin*=154 bp)

- Muscle

human Nag-1

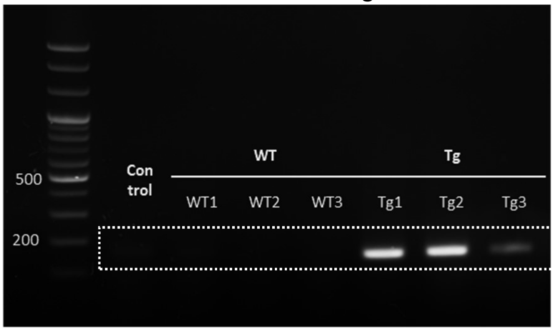

β-actin

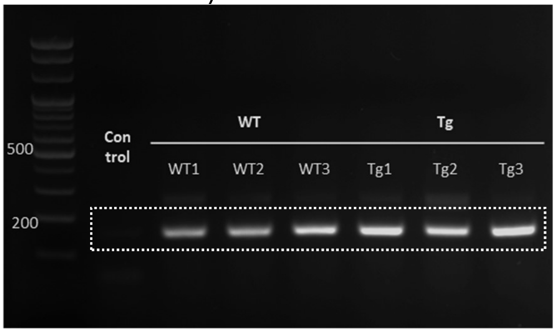

- iWAT

human Nag-1

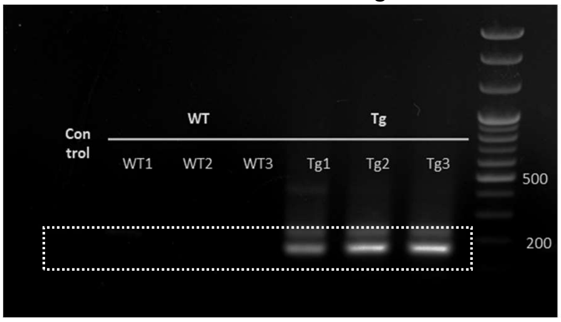

β-actin

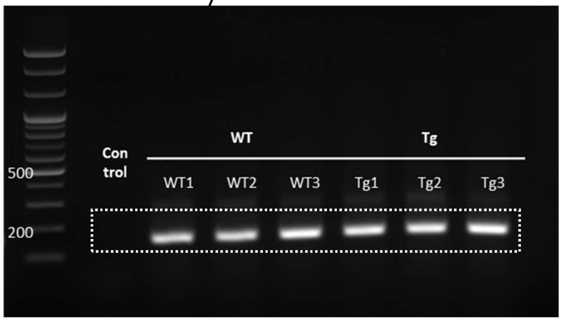

- BAT

human Nag-1

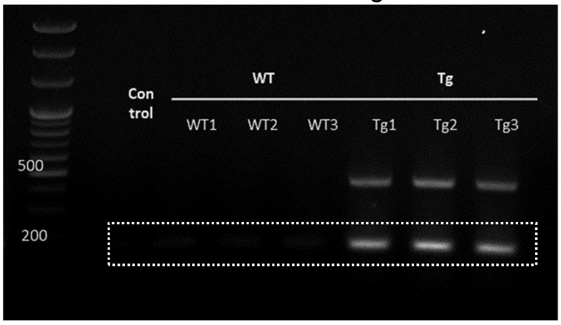

β-actin

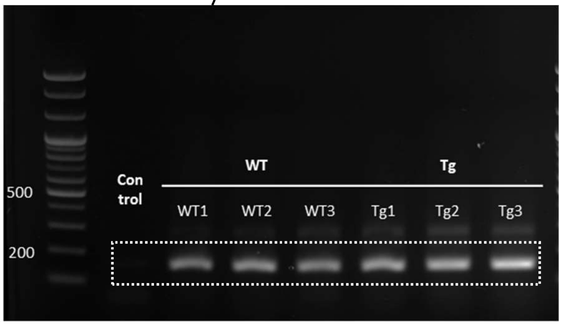

Supplementary Figure 5. The expression of p-Akt, human NAG-1 and β-actin protein in muscle using western blot.

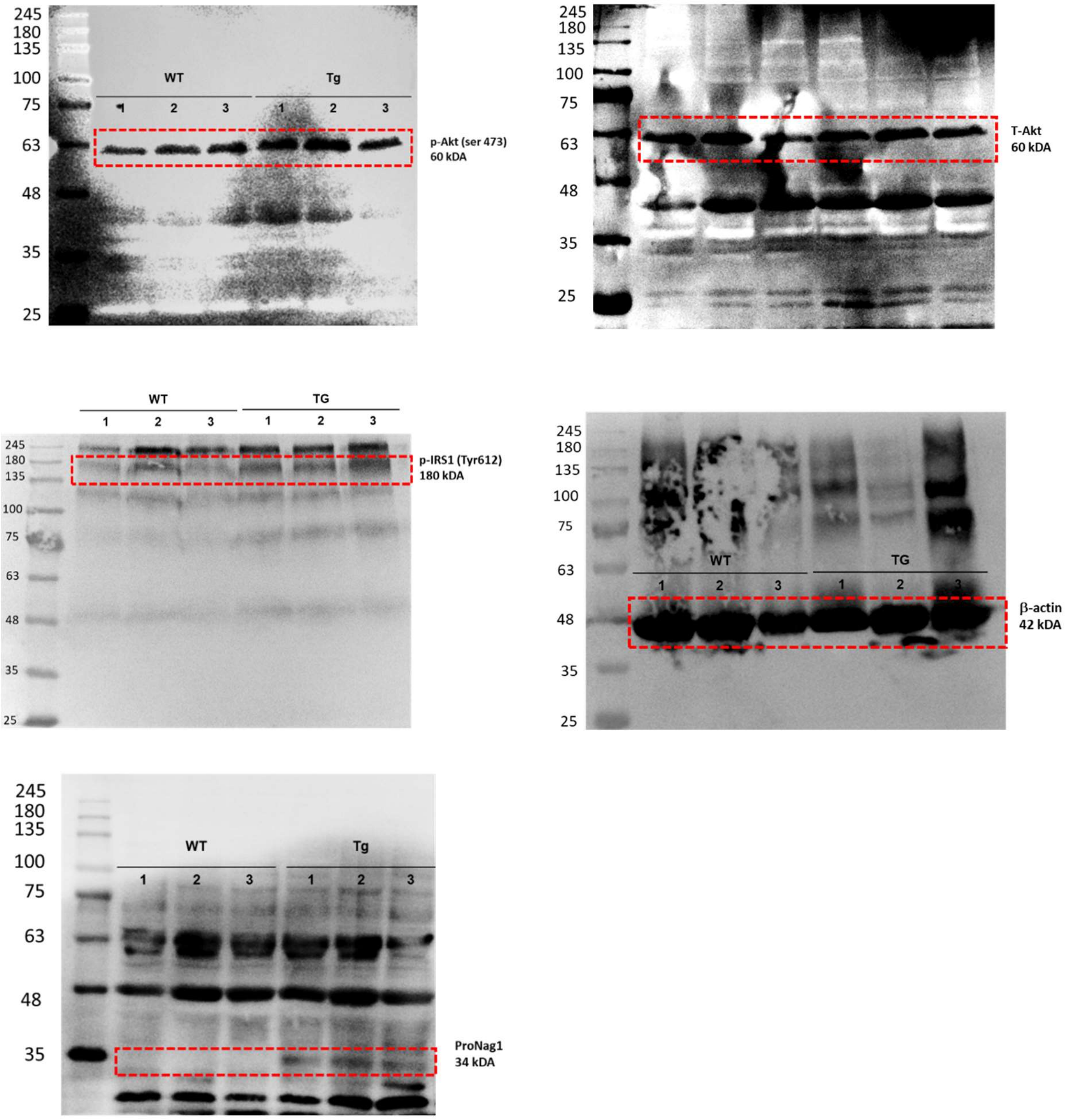

*Parallel membrane
